# Dopamine D2 Receptor Activation Blocks GluA2/ROS Positive Feedback Loop to Alienate Chronic-Migraine-Associated Pain Sensitization

**DOI:** 10.3390/antiox13060725

**Published:** 2024-06-14

**Authors:** Wei Zhang, Xiaoyan Zhang, Ming Lei, Dunke Zhang, Guangcheng Qin, Jiying Zhou, Lichun Ji, Lixue Chen

**Affiliations:** 1 Laboratory Research Center, The First Affiliated Hospital of Chongqing Medical University, Chongqing 400016, China; 2023130059@stu.cqmu.edu.cn (W.Z.); 203396@hospital.cqmu.edu.cn (G.Q.); 2 Department of Neurology, The First Affiliated Hospital of Chongqing Medical University, Chongqing 400042, China; zhangxiaoyan678@sina.cn (X.Z.); 201619@hospital.cqmu.edu.cn (J.Z.); 3 Department of Respiration, The Thirteenth People’s Hospital of Chongqing, Chongqing 400016, China

**Keywords:** pain sensitization, GluA2, dopamine D2 receptor, mitochondrial calcium overload, oxidative stress

## Abstract

Chronic migraine is a disabling disorder without effective therapeutic medicine. AMPA receptors have been proven to be essential to pathological pain and headaches, but the related regulatory mechanisms in chronic migraine have not yet been explored. In this study, we found that the level of surface GluA2 was reduced in chronic migraine rats. Tat-GluR23Y (a GluA2 endocytosis inhibitor) reduced calcium inward flow and weakened synaptic structures, thus alleviating migraine-like pain sensitization. In addition, the inhibition of GluA2 endocytosis reduced the calcium influx and alleviated mitochondrial calcium overload and ROS generation in primary neurons. Furthermore, our results showed that ROS can induce allodynia and GluA2 endocytosis in rats, thus promoting migraine-like pain sensitization. In our previous study, the dopamine D2 receptor was identified as a potential target in the treatment of chronic migraine, and here we found that dopamine D2 receptor activation suppressed chronic-migraine-related pain sensitization through blocking the GluA2/ROS positive feedback loop in vivo and in vitro. Additionally, ligustrazine, a core component of *ligusticum chuanxiong*, was shown to target the dopamine D2 receptor, thereby alleviating ROS production and abnormal nociception in CM rats. This study provides valuable insight into the treatment of chronic migraine.

## 1. Introduction

Chronic migraine (CM) is a primary neurological disorder that lacks effective treatment due to its uncertain mechanism [1]. Central sensitization, caused by the excessive activation of the nociceptive transmission system, serves as the primary pathogenesis of chronic migraine, clinically manifesting as cutaneous allodynia [2,3]. Evidence suggests that oxidative stress and mitochondrial function play important roles in chronic migraine [4,5]. Nevertheless, the upstream regulatory mechanisms of mitochondrial oxidative stress and how oxidative stress regulates central sensitization in CM remain to be explored.

Growing evidence shows that the α-Amino-3-hydroxy-5-methyl-4-isoxazole propionic acid receptors (AMPARs) play a crucial role in chronic pain [6,7], and they were proven to mediate the initial rapid transmission of injurious messages in the primary sensory neurons and facilitate central sensitization in neuropathic pain [8,9]. AMPARs are composed of four subunits (GluA1-4), of which GluA1-containing AMPAR is Ca^2+^-permeable while GluA2-containing AMPAR is Ca^2+^-impermeable [10,11]. Our previous study proved that GluA1 externalization contributes heavily to pain sensitization in CM [12]. It is reported that pharmacological inhibition of GluA2 internalization relieves neuropathic pain [13], but the role of GluA2 has not been explored in chronic migraine.

As a class of calcium channels, GluA2 synergistically controls intracellular calcium concentrations and permits a continuous influx of calcium during pain sensitization. Excessive calcium concentrations were proven to mediate mitochondrial calcium overload, impairing mitochondrial function and contributing to the release of excessive mitochondrial reactive oxygen species (ROS). Moreover, a few studies reported that ROS overproduction regulates the synaptic location of the AMPARs [14]; this, in turn, promotes the sensitization of dorsal horn neurons and persistent pain [15]. However, whether the GluA2 internalization/ROS production loop participates in central sensitization in CM remains unclear.

Dopamine receptors can be classified into two groups: the D1 family (D1R and D5R) and the D2 family (D2R–D4R) [16,17]. Of these, D1R and D2R are the predominant receptors in the Central Nervous System (CNS) [17]. Recent studies showed that D1R and D2R differentially modulate neuropathic pain [18], and our previous study proved that D2R, but not D1R, exerts an anti-injury effect in the trigeminal nucleus caudalis (TNC) in CM [12]. In addition, it has been reported that D2R can modulate AMPAR-mediated neuroexcitatory toxicity and alter the distribution of AMPAR receptors on synaptic membranes [19]. Most importantly, D2R was reported to play both ROS-chelating and anti-oxidative-stress roles [20]. 

Traditional Chinese medicine (TCM) has a long history of treating migraine, and a variety of herbal components have shown great potential for relieving chronic pain [21,22], including ligustrazine, the main active ingredient of *ligusticum chuanxiong hort*, which reportedly attenuates the production of ROS and resists oxidative stress in a variety of neurological disorders [23,24,25]. However, whether ligustrazine can target D2R to alienate chronic migraine remains unclear. Therefore, we proposed the following hypothesis: that D2R can be the target of ligustrazine and regulate the GluA2 internalization/mitochondrial calcium overload/ROS overgeneration loop to regulate pain sensitization in CM.

In this research, we found that GluA2 endocytosis inhibition reduced mitochondrial calcium overload and ROS release in chronic migraine model rats. Importantly, we confirmed that D2R activation can block the GluA2/ROS feedback loop to alleviate pain sensitization in CM, and we identified ligustrazine as a possible upstream regulator of D2R in antioxidative stress in CM. Our research emphasizes the important effect of antioxidative treatment in CM, suggesting that D2R could serve as an effective target for CM.

## 2. Materials and Methods

### 2.1. Animals

To avoid the potential interaction of estrogen signaling and the dopamine system [26,27], a total of 132 male SD rats were chosen for the experiments (specific-pathogen-free rats, provided by Chongqing Medical University). For primary neuron isolation, pregnant rats (Day 18) were chosen. Rats were housed in a standard environment (24 ± 1 °C; a 12 h light/dark cycle) with no limit to food and water acquisition. The experimenters were blinded to the randomized animal experiments.

### 2.2. Materials

The inflammatory soup (IS) information is listed in Table 1, and the antibodies used in this work are listed in Table 2. Quinpirole was provided by Sigma-Aldrich (St. Louis, MO, USA); Tat-GluR23y and Scrambled peptide were provided by AnaSpec (Fremont, CA, USA). PBN, sulpiride, and ligustrazine were purchased from MedChemExpress (Shanghai, China), and t-BHP was provided by Macklin (Shanghai, China); L-AMPA was purchased from Tocris (Minneapolis, MN, USA). Fluo4 AM, JC-1, and DHE probe were provided by Beyotime (Haimen, China); Rhod-2 AM was purchased from Yeason (Shanghai, China); a phosphatase inhibitor cocktail was obtained from MedChemExpress (Shanghai, China); RIPA buffer and PMSF were provided by Beyotime (Haimen, China).

### 2.3. Surgery and Chronic Migraine Models

The specific protocol has been described previously [12]. Briefly, rats were deeply anesthetized with isoflurane and held on a stereotaxic frame (Stoelting, Wood Dale, IL, USA); then, an incision was created to expose the bregma point. Next, a craniotomy was made using a drill according to the corresponding coordinate (1.0 mm to the left, 1.5 mm to the side of the bregma) where a stainless-steel cannula was fixed using dental cement. The rats recovered for one week after the surgery. For chronic migraine induction, the “Inflammatory soup” was dripped through the sterile catheter onto the dura mater of the rats for 7 days. 

### 2.4. Pain Threshold Test

The pain thresholds were tested in animals of different groups to evaluate allodynia and central sensitization, and the specific steps have been described in previous work [12]. Briefly, the rats were placed in special cages for 30 min for acclimation, and von Frey filaments were applied using the “up-down” method to quantify the plantar or periorbital mechanical pain threshold; specifically, von Frey filaments were used to stimulate the hind paw foot surface five times, with 1 min intervals between each stimulus, and it was considered significant if there were three or more positive responses (such as paw lifting and licking) out of five stimulations. The rat plantar area was stimulated using continuous radiant heat to quantify the thermal pain thresholds. The above tests were performed once before surgery to exclude rats with abnormal basal pain thresholds.

### 2.5. Drug Administration

**Figure 1:**
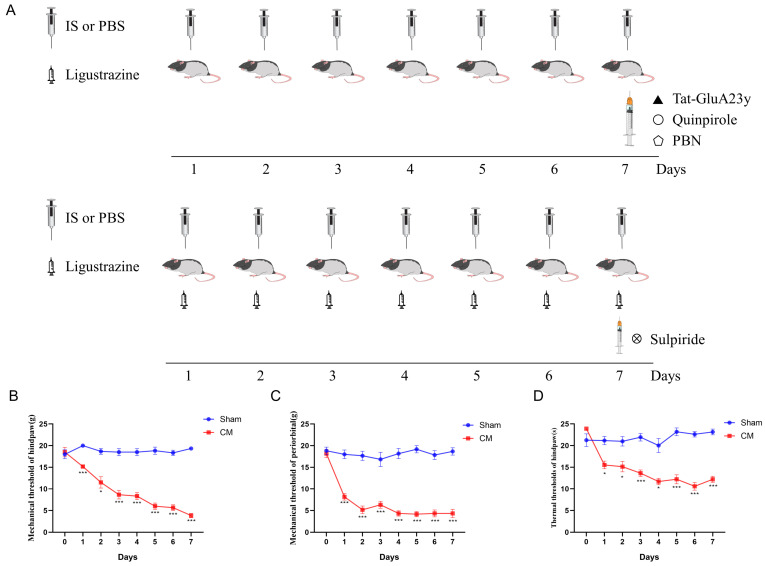
Characterization of chronic migraine models. (**A**) The timeline of animal experiments. (**B**–**D**) The plantar mechanical pain thresholds, thermal pain thresholds, and periorbital pain thresholds were markedly downregulated in the CM group. Data are the mean ± SEM (*n* = 6/group). * *p* < 0.05, *** *p* < 0.001 vs. the Sham group.

The IS (5 μL) was dripped onto the dura of the rats for 7 days to establish CM rat models, and the IS was replaced with PBS in Sham rats. To test the effect of D2R in CM rats, quinpirole (10 μg, dissolved in PBS) was injected into CM rats [12]; to test the effect of GluA2 on CM rats, Tat-GluR23y (10 μL, 10 μM dissolved in 5% DMSO) [13] was administered to CM rats. All relative drugs were administered in the lateral ventricle through the fixed cannula (1.0 mm to the left, 1.5 mm to the side of the bregma, 4.0 mm in depth). t-BHP (the ROS donor, 100 mg/kg, i.p.) and PBN (the ROS chelator, 150 mg/kg, i.p.) were applied to investigate the role of ROS in CM. In the ligustrazine + sulpiride group, ligustrazine was delivered via gastric administration (i.g.) synchronized with the IS drip, and sulpiride was injected on Day 7 after the pain threshold test. The control group was treated with 5% DMSO. The doses of all drugs were selected based on previous studies. A schematic diagram of the treatments applied in the animal experiments is shown in Figure 1A.

### 2.6. Protein Extraction and Western Blot

TNC tissues were lysed with RIPA buffer (with 1 mM PMSF and 1 mM phosphatase inhibitor cocktail) to extract the total protein. For the isolation of plasma membrane fractions, a Membrane and Cytosol Protein Extraction Kit (Beyotime, Haimen, China) was applied. In brief, tissues were moderately lysed by homogenization, the nucleus and some precipitates were removed by low-speed centrifugation, and the cell membrane precipitates were obtained by high-speed centrifugation. The membrane proteins were obtained from the precipitate using the optimized membrane protein extraction reagent in this kit. The expression of plasma membrane GluA2 (represented by m-GluA2) was detected by Western blotting to assess GluA2 internalization. For Western blotting, the isolated protein was separated by an SDS-PAGE gel and transferred onto PVDF membranes. After blocking with 5% non-fat milk at RT for 1.5 h, the membranes were incubated with the primary antibodies overnight at 4 °C. The next day, the membranes were exposed using an imaging system with an ECL reagent (ZEN-BIOSCIENCE, Chengdu, China) after incubation with the second antibody. For the total proteins, GAPDH was used for normalization. For the membrane proteins, N-cadherin was used for normalization.

### 2.7. Immunofluorescence Staining

Deeply pentobarbital anesthetized rats were cardiac-infused with 4% paraformaldehyde (PFA) to obtain intact TNCs. TNCs were cut into 15 μm slices using a cryostat (Leica, Wetzlar, Germany) after dehydration and post-fixation. The sections were then penetrated by Triton (3% in PBS) and blocked with 10% goat serum (Boster, Wuhan, China) for 1 h at 37 °C. After incubation with the primary antibodies (overnight; 4 °C) and the secondary antibodies (1 h; 37 °C), the sections were stained using DAPI for 10 min at 37 °C and sealed with glycerin. A confocal microscope was applied to collect the pictures. Data were analyzed using Image J (version 1.54f).

### 2.8. Primary Neuron Culture

Brainstems containing TNCs were isolated from embryos (Day 18) and digested with papain for 30 min at 37 °C; these were then repeatedly blown to obtain cell suspensions, which were subsequently centrifuged and resuspended with DMEM-HG medium with 10% fetal bovine serum. Finally, cells were plated on poly-L-lysine-coated confocal dishes and cultured at 37 °C with 5% CO_2_. After 6 h, the DMEM-HG medium was replaced with Neurobasal medium containing 2% B27 and L-glutamine (0.05 mg/mL). All the steps were performed in a sterile environment.

### 2.9. Monitoring of Ca^2+^ Concentration

Fluo4 AM was applied to detect the intracellular Ca^2+^ content. The specific procedures were described previously [12]. In brief, neurons cultured on Day 7 were incubated with Fluo4 AM working solution (4 μM, dissolved in PBS) for 0.5 h at 37 °C and 5% CO_2_. After washing with HBSS, neurons were stimulated by AMPA (50 μM) and visualized using a confocal microscope for a total of 5 min (capturing one picture every 5 s).

### 2.10. Mitochondrial Ca^2+^ Detection

For mitochondrial calcium analysis, neurons were first incubated with Rhod-2 AM for 30 min at 37 °C; after washing with HBSS, the neurons were subsequently incubated with Mitotracker-green for 30 min at 37 °C. After staining with Hoechst, neurons were visualized using a Confocal microscope (Zeiss, Jena, Germany). The JACOP plugin of ImageJ software (version 1.54f) was used to analyze the co-localization of mitochondria and Ca^2+^, and the Pearson’s value was applied to describe the mitochondrial Ca^2+^ concentration.

### 2.11. JC-1 and ROS Staining

For JC-1 staining, neurons were incubated with the JC-1 probe for 30 min at 37 °C according to the manufacturer’s instruction and were observed using fluorescence microscopy (Olympus, Tokyo, Japan). The DHE or H2DCFDA probe was applied to detect ROS levels; briefly, neurons were incubated with the probe for 30 min at 37 °C. After staining with Hoechst, the neurons were observed using fluorescence microscopy (Olympus, Tokyo, Japan). Pictures were analyzed using ImageJ software.

### 2.12. Molecular Docking

Molecular docking was performed to predict the possible interaction of receptors and protein ligands by AutoDock 4.2 software. The structure of the D2R protein (ID: 6VMS) was downloaded from the *Protein Data Bank* (https://www.rcsb.org/, accessed on 24 June 2023). The information on ligustrazine molecular (ID: 14296) was obtained from *PubChem* (https://pubchem.ncbi.nlm.nih.gov/, accessed on 24 June 2023). After removing water and adding hydrogen, the D2R protein and ligustrazine molecule were exported into the “*pdbqt”* format for molecular docking. The AutoDockTools 1.5.7 package was used to perform molecular docking and analyze the optimal bonding energy. The binding structures were generated and output by PyMOL software (Open Source, Version 2.2.0, accessed on 6 August 2023).

### 2.13. Statistical Analysis

All data are the mean ± SEM in this study. Two-way analysis of variance (ANOVA) with a Bonferroni post hoc test was used to analyze the pain behavioral data. Multiple comparisons were carried out by one-way ANOVA with a Bonferroni post hoc test. Two-group comparisons were performed by the unpaired *t*-test. *p* < 0.05 was considered statistically significant. All the graphs were created by GraphPad Prism 8.

## 3. Results

### 3.1. Pharmacological Blockade of GluA2 Endocytosis Alleviated Chronic Migraine

**Figure 2:**
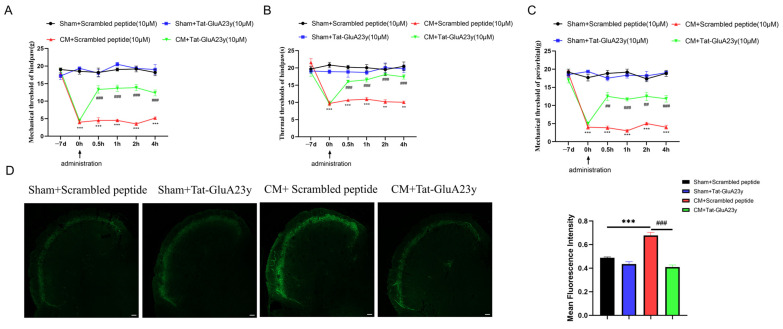
Inhibition of GluA2 endocytosis relieved migraine-like pain. (**A**–**C**) Tat-GluA23y injection alleviated the mechanical, thermal, and periorbital pain hypersensitivity. (**D**) Tat-GluA23y decreased CGRP expression in CM rats, as shown by immunofluorescence. Scale bar = 200 μm. Data are the mean ± SEM (*n* = 6/group). ** *p* < 0.01, *** *p* < 0.001 vs. the Sham+Scrambled peptide group; ## *p* < 0.01, ### *p* < 0.001 vs. the CM+Scrambled group.

First, the plantar (mechanical and thermal) and periorbital pain thresholds were measured to ensure the validity of the CM models; the decrease in pain thresholds suggested that the CM rats were successfully established (Figure 1B–D). Next, Tat-GluA23y (a GluA2 internalization inhibitor) was used to understand the role of GluA2 in central sensitization in chronic migraine. The results suggested that Tat-GluA2-3y injection markedly alleviated allodynia in CM rats (Figure 2A–C). In addition, the fluorescence intensity of CGRP, the biological marker of migraine, was sharply lowered by the Tat-GluA23y treatment (Figure 2D), indicating that the GluA2 endocytosis blockade relieved chronic-migraine-like pain in CM rats. 

### 3.2. GluA2 Endocytosis Inhibition Reduced Mitochondrial Calcium Overload and ROS Production

**Figure 3:**
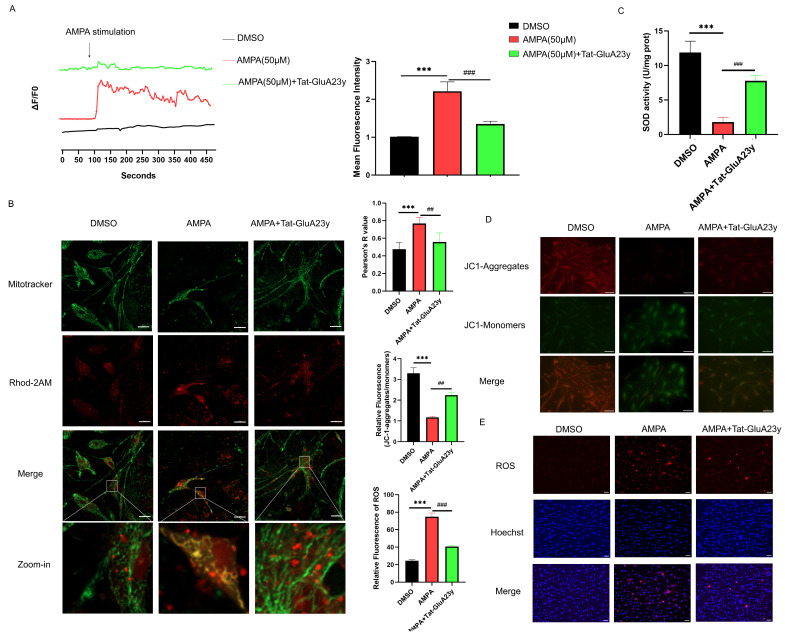
Inhibition of GluA2 endocytosis alleviated mitochondrial calcium overload and ROS burst. (**A**) Traces of calcium inward flow in primary neurons cultured in vitro among groups. (**B**) Tat-GluA23y pre-incubation alleviated mitochondrial calcium overload induced by L-AMPA, as shown by Mitotracker-green and Rhod-2 AM double staining. Scale bar = 20 μm. (**C**) Tat-GluA23y reversed the reduced SOD activity induced by L-AMPA. (**D**) Tat-GluA23y reversed the decreased mitochondrial membrane potential induced by AMPA, as shown by JC-1 staining. Scale bar = 20 μm. (**E**) Tat-GluA23y decreased the ROS overproduction induced by AMPA, as shown by DHE staining. Scale bar = 50 μm. Data are the mean ± SEM (*n* = 3/group). *** *p* < 0.001 vs. the DMSO group; ## *p* < 0.01, ### *p* < 0.001 vs. the L-AMPA group.

Next, we further investigated the role of GluA2 in intra-neuronal calcium concentrations. Interestingly, Tat-GluA23y application significantly weakened the calcium inward flow stimulated by L-AMPA (which can increase the neuronal calcium concentration) in TNC-contained primary neurons cultured in vitro (Figure 3A). It was previously reported that an increased intracellular calcium concentration can lead to mitochondrial calcium overload and dysfunction. We then detected the mitochondrial calcium concentration and SOD activity, and the results showed that mitochondrial calcium uptake was increased, represented by increased calcium fluorescence and mitochondrial co-localization after L-AMPA stimulation; this increase was reversed by Tat-GluA23y (Figure 3B). The SOD activity was decreased by L-AMPA; this decrease was, in turn, reversed by Tat-GluA23y pre-incubation (Figure 3C). JC-1 staining suggested that Tat-GluA23y could protect the impaired mitochondrial function from L-AMPA stimulation (Figure 3D). Likewise, the DHE staining also suggested that AMPA boosted the ROS output that was inhibited by Tat-GluA23y (Figure 3E). The above results indicate that the inhibition of GluA2 internalization can protect mitochondria from L-AMPA-mediated calcium overload.

### 3.3. ROS Regulated Migraine-like Allodynia and GluA2 Trafficking

**Figure 4:**
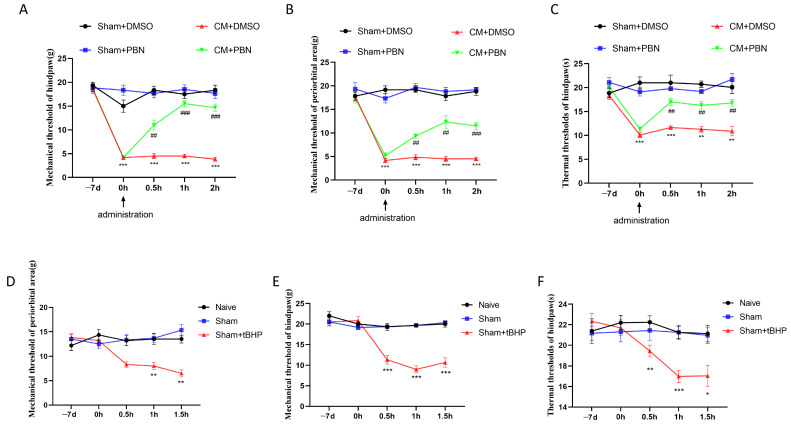
ROS regulated migraine-like pain hypersensitivity in rats. (**A**–**C**) PBN injection raised the mechanical, thermal, and periorbital pain thresholds in CM rats. Data are mean ± SEM (*n* = 6/group). ** *p* < 0.01, *** *p* < 0.001 vs. the Sham+DMSO group; ## *p* < 0.01, ### *p* < 0.001 vs. the CM+DMSO group. (**D**–**F**) tBHP administration reduced the mechanical, thermal, and periorbital pain thresholds in Sham rats. Data are the mean ± SEM (*n* = 6/group). * *p* < 0.05, ** *p* < 0.01, *** *p* < 0.001 vs. the Sham group.

**Figure 5:**
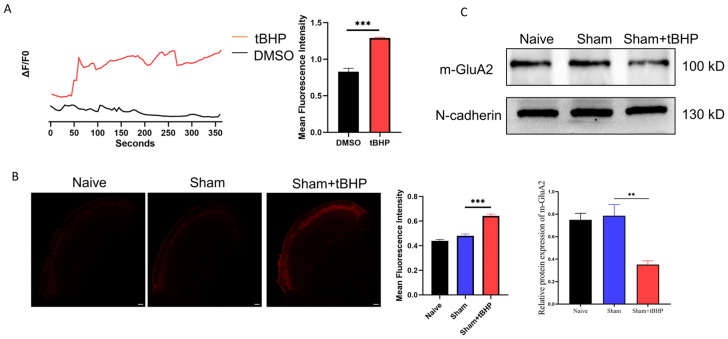
ROS regulated calcium influx in vitro and GluA2 endocytosis in CM rats. (**A**) Traces of calcium inward flow in primary neurons cultured in vitro among groups. Data are the mean ± SEM (*n* = 10 cells/group). *** *p* < 0.001 vs. the DMSO group. (**B**) tBHP injection elevated CGRP levels in the TNC in Sham rats. Scale bar = 200 μm. (**C**) tBHP injection increased the membrane level of GluA2 in CM rats. Data are the mean ± SEM (*n* = 6/group). ** *p* < 0.01, *** *p* < 0.001 vs. the Sham group.

ROS are reported to regulate pain behaviors and AMPARs’ function; therefore, PBN, an ROS chelator, was applied to further explore the role of ROS in GluA2 endocytosis and CM-related pain sensitization. As shown in Figure 4A–C, PBN, but not DMSO administration, raised the pain thresholds of CM rats. Subsequently, tert-Butyl hydroperoxide (tBHP), an ROS donor, was administered to the Sham rats to fully understand the role of ROS in GluA2 endocytosis and allodynia. As shown in Figure 4D–F, tBHP administration decreased the pain thresholds of the Sham rats. These results suggest that ROS participate in the regulation of migraine-like pain sensitization. A subsequent analysis revealed that tBHP promoted calcium inward flow in primary neurons (Figure 5A). Moreover, tBHP upregulated the expression of CGRP, as shown by immunofluorescence (Figure 5B).

ROS are reported to play a role in AMPAR trafficking [28,29], and we found that tBHP administration markedly increased the synaptic level of GluA2 in rats, as detected by Western blotting (Figure 5C). 

### 3.4. D2R Activation Intercepted the GluA2/ROS Feedback Loop

**Figure 6:**
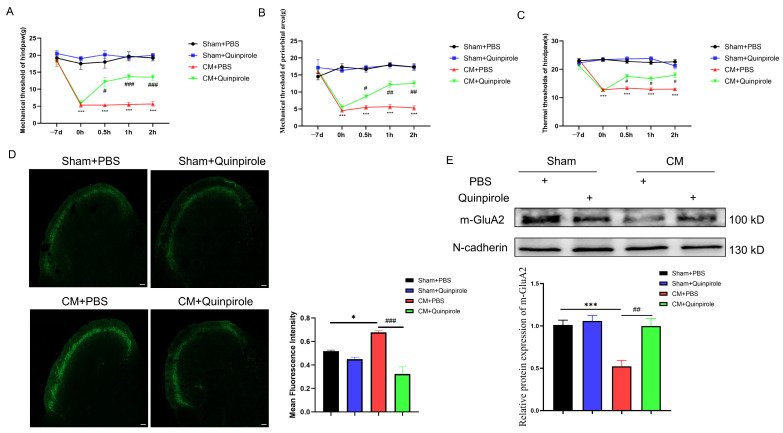
D2R activation relieved pain sensitization and GluA2 endocytosis in CM rats. (**A**–**C**) Quinpirole injection elevated pain thresholds in CM rats. (**D**) Quinpirole injection reduced CGRP expression. Scale bar = 200 μm. (**E**) Quinpirole injection reduced GluA2 endocytosis, as shown by Western blotting. Data are the mean ± SEM (*n* = 6/group). * *p* < 0.05, *** *p* < 0.001 vs. the Sham+PBS group; # *p* < 0.05, ## *p* < 0.01, ### *p* < 0.001 vs. the CM+PBS group.

Our previous work suggested that D2R can regulate AMPAR trafficking in chronic migraine [12]. Thus, we next examined the role of D2R in GluA2 internalization and ROS production. First, the impact of quinpirole, the D2R agonist, on pain hypersensitivity was tested. The results revealed that quinpirole, but not DMSO, alleviated allodynia in CM rats (Figure 6A–C). The subsequent results showed that quinpirole inhibited elevated CGRP expression in CM rats (Figure 6D). Importantly, D2R activation significantly increased the membrane expression of GluA2, as shown by Western blotting (Figure 6E).

**Figure 7:**
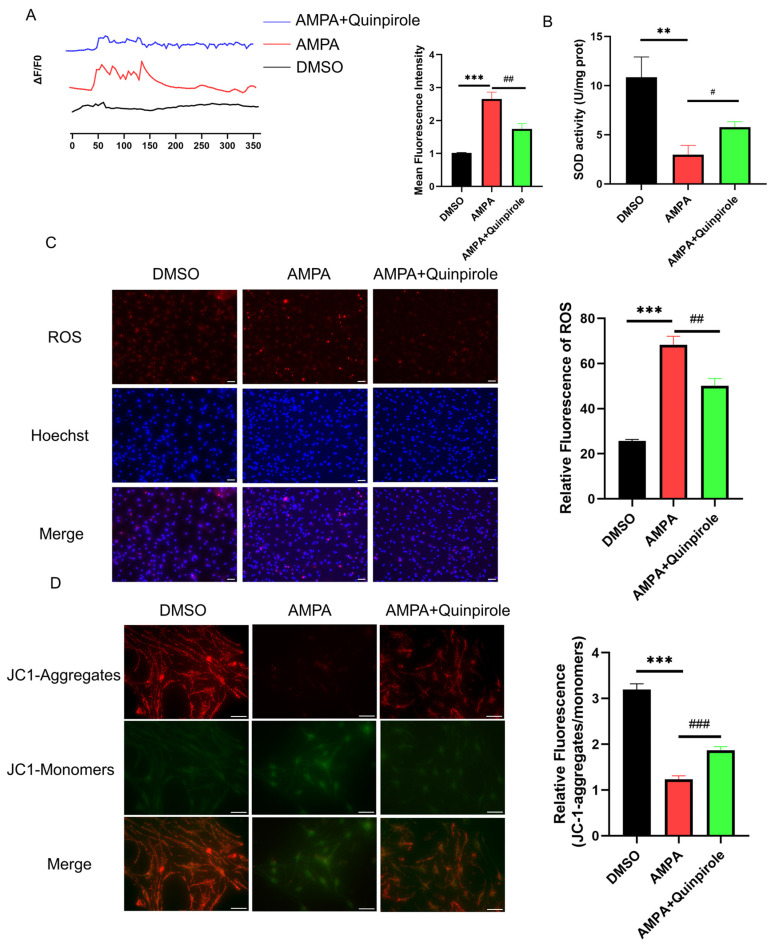
D2R activation relieved calcium influx and oxidative stress in vitro. (**A**) Traces of calcium inward flow in primary neurons cultured in vitro among groups. Data are the mean ± SEM (*n* = 10 cells/group) *** *p* < 0.001 vs. the DMSO group; ## *p* < 0.01 vs. the L-AMPA group. (**B**) Quinpirole reversed the SOD activity decrease induced by L-AMPA. (**C**) Quinpirole prevented the ROS overproduction induced by L-AMPA, as shown by DHE staining. Scale bar = 50 μm. (**D**) Quinpirole reversed the decreased mitochondrial membrane potential induced by L-AMPA, as shown by JC-1 staining. Scale bar = 20 μm. Data are the mean ± SEM (*n* = 3/group). ** *p* < 0.01, *** *p* < 0.001 vs. the DMSO group; # *p* < 0.05, ## *p* < 0.01, ### *p* < 0.001 vs. the L-AMPA group.

Next, we examined the effect of quinpirole on Ca^2+^ influx in vitro. As shown in Figure 7A, quinpirole pre-incubation effectively decreased the intracellular calcium concentration stimulation by L-AMPA. Interestingly, quinpirole inhibited mitochondrial ROS burst, elevated SOD activity after its impairment by L-AMPA (Figure 7B,C), and rescued the impaired mitochondrial membrane potential, as shown by JC-1 staining (Figure 7D). The above results suggest that D2R activation blocks the ROS/GluA2 loop and may serve as a molecular target for CM treatment.

### 3.5. D2R May Be a Molecular Target of Ligustrazine for Treating CM

**Figure 8:**
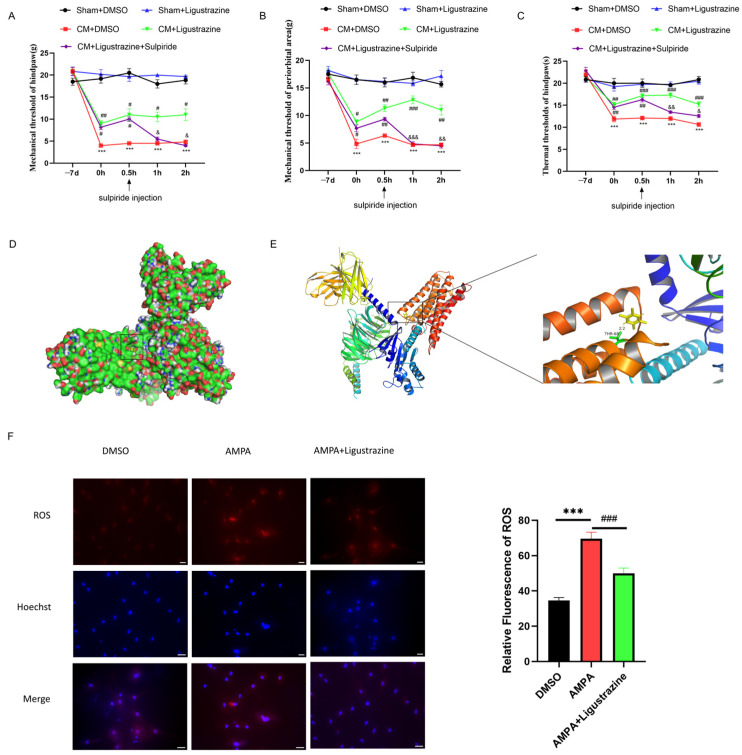
Ligustrazine can target D2R and regulate allodynia and ROS production. (**A**–**C**) Ligustrazine relieved pain hypersensitivity and was offset by sulpiride. Data are mean ± SEM (*n* = 6/group). *** *p* < 0.001 vs. the Sham+DMSO group; # *p* < 0.05, ## *p* < 0.01, ### *p* < 0.001 vs. the CM+DMSO group; & *p* < 0.05, && *p* < 0.01, &&& *p* < 0.001 vs. the CM+Ligustrazine group. (**D**) The 3D structure of the ligustrazine–D2R complex. (**E**) The potential binding sites between D2R and ligustrazine. Ligustrazine is marked in yellow, and hydrogen bonds are marked in green. (**F**) Ligustrazine prevented the ROS burst induced by L-AMPA in primary neurons in vitro. Scale bar = 50 μm. Data are the mean ± SEM (*n* = 3/group).*** *p* < 0.001 vs. the DMSO group; ### *p* < 0.001 vs. the L-AMPA group.

To promote the clinical relevance of D2R as a therapeutic target for CM, we set our sights on traditional Chinese medicines that have been used for hundreds of years to treat migraine in China. Of these, *chuanxiong hort* is one of the most famous, with ligustrazine having been proven as its main active ingredient. Hence, the impact of ligustrazine on pain hypersensitivity was tested, and the results revealed that ligustrazine, but not DMSO, alleviated allodynia in CM rats (Figure 8A–C). Next, to verify the possibility of ligustrazine and D2R integration, molecular docking of ligustrazine on the D2R protein was performed using AutoDock 4.2. The results suggest that D2R has a binding site with ligustrazine (Figure 8D,E), and the best binding energy value was −4.24 kcal/mol (Table 3). Interestingly, ligustrazine markedly blocked ROS overproduction in vitro (Figure 8F). However, under normal conditions, neither ligustrazine nor quinpirole treatment affected neuronal ROS production or the mitochondrial membrane potential (Supplementary Figure S1A,B). Furthermore, additional data showed that ligustrazine and quinpirole did not show significant differences in their ROS scavenging ability (Supplementary Figure S1C).

The above results confirmed the palliative effect of ligustrazine on CM-related pain sensitization, suggesting that medicines targeting D2R may have great potential in CM treatment.

## 4. Discussion

The main purpose of this research was to explore the roles of GluA2 and D2R in chronic-migraine-related pain sensitization; the principal findings are as follows: (1) a blockade of GluA2 internalization alleviated mitochondrial calcium overload, ROS burst, and migraine-like pain; (2) ROS/GluA2 endocytosis contributed to CM-related pain sensitization; and (3) D2R regulated the ROS/GluA2 internalization loop and pain hypersensitivity, serving as a possible pharmacological target of ligustrazine.

The chronification of migraine is attributable to a persistent enhancement of synaptic plasticity in the trigeminal system, also known as central sensitization [14,15]. As reported previously, AMPARs and NMDARs contribute heavily to synaptic plasticity progression [30,31]. Growing evidence confirms that the progressive accumulation of GluA1-containing AMPAR on the synaptic surface at the onset of synaptic enhancement permits the persistent transmission of injurious information due to its permeability to calcium [32]. AMPAR-mediated calcium influx can activate the NR2B subunit of NMDARs, removing the magnesium that impedes NMDAR-mediated calcium inward flow and mediating a large and persistent calcium influx, which induces pain sensitization [6,33].

However, one limitation of this study is the exclusion of female mice. This is because women are more likely to experience chronic migraines, which have been reported to be possibly related to estrogen flushes. The dopamine system has been reported to crosstalk with the estrogen system, and there are sex differences in dopamine receptors in pain regulation [34,35]. It has been reported that changes in estrogen levels can modulate dopamine receptor expression [36]; therefore, gender differences in the dopamine D2 receptor modulation of chronic migraine remain to be further investigated in subsequent studies.

Unlike GluA1-containing AMPAR, GluA2-containing AMPAR is impermeable to calcium [37], and several studies have confirmed that GluA2 plays a critical role in neuropathic pain [38,39]. In our study, we found that the inhibition of GluA2 endocytosis alleviated migraine-like pain behavior in CM rats, suggesting that GluA2 regulates the structural strength and message transmission of synapses and contributes to the chronification of migraine. However, AMPARs frequently form GluA1/A2 and GluA2/A3 multimers at the postsynaptic membrane through different combinatorial patterns [40], the specific distribution patterns of which were not investigated in this research. Determining the AMPAR subunit composition on the synaptic membrane in CM could be the next task in future research.

Intracellular calcium is essential for maintaining normal neuronal physiological activity, but, during pain sensitization, neuronal calcium channels allow more calcium to enter, facilitating neuronal electrical conduction [41,42]. However, calcium accumulation may contribute to increased mitochondrial calcium, which, in turn, may lead to mitochondrial dysfunction, one manifestation of which is the output of ROS [43]. Several studies have reported that ROS have a regulatory effect on glutamate receptors, including AMPARs and NMDARs, and synaptic transmission [15,44]. In the present study, our results suggest that enhanced GluA2 endocytosis leads to an increased calcium content and mitochondrial calcium overload, resulting in mitochondrial dysfunction and ROS burst. This, in turn, promotes GluA2 internalization. Additional results showed that ROS mediated the enhancement of dendritic spine density, a hallmark of pain sensitization.

The trafficking of AMPARs is regulated by several factors, such as glutamate receptor-interacting protein (GRIP) [45], miRNAs [46], and epigenetic modulators [47], and it requires some different auxiliary subunits [10,48]. D2R was characterized as a potential upstream regulator of GluA2 internalization in this study, and quinpirole was shown to increase the membrane expression of GluA2. As a G-protein-coupled receptor, D2R shows various downstream signaling activities and participates in a large number of physiological and pathological progressions [16,49]. Our previous study revealed that the PI3K pathway and Src kinase family acted as bridges between D2R and GluA1-containing AMPAR trafficking [12]. Unlike the PI3K or Src kinase family, PKA receives the direct regulation of D2R and was confirmed to mediate GluA2 trafficking [29]. However, some non-G-protein-coupled receptor-regulated proteins are also involved in the regulation of AMPAR by D2R. Zou et al. reported that D2R can interact with NSF to exert a transport regulatory function on AMPARs [19]. Notably, D2R has been reported to have mitochondrial protective and antioxidant effects [50], but whether D2R signaling is directly associated with mitochondrial components remains unclear. Thus, other potential mediators, such as mitochondrial components or the auxiliary subunits involved, need to be further determined in future research. 

In this work, quinpirole was used as a D2R agonist, and it is reported that quinpirole can also be applied as a dopamine D3 receptor (D3R) agonist. Ignoring the agonistic effect of quinpirole on D3R is a flaw in this study. D3R is a member of the D2-like receptor family and potentially interacts or co-operates with D2R [51]. An imbalance between D2R and D3R in migraine patients has been reported [52]; hence, the role of D3R in migraine cannot be ignored. Furthermore, the place occupied by D3R in pain relief by D2R/D3R agonists (such as quinpirole) remains unclear. It is reported that quinpirole has a Ki value of 4.8 nM for D2R and 5.1 nM for D3R, suggesting that quinpirole has a similar affinity for D2R and D3R [53]. However, further results showed that quinpirole primarily binds to D2R by using [3H]quinpirole, whereas the guanine nucleotide-insensitive component of [3H]quinpirole (about 30%) binds to D3R [53].

Studies have shown the potential role of D3R in pain regulation [54]. D3R is mainly distributed in the thalamus and cortex. In addition, the expression of D3R is lower than that of D2R at the spinal cord level [55], suggesting that D2R occupies a more dominant role in analgesia compared with D3R. However, there is still a lack of distribution of D3R in the trigeminal system, as well as a lack of clarity regarding whether D2R or D3R is more predominant in the trigeminal system. Therefore, it is necessary to further explore the role of D3R in chronic migraine. 

Botanical herbs have been used to treat migraine in China for a long time, and some traditional Chinese medicines have been widely proven to have good efficacy and very few side effects in treating migraine [21,22]. In recent studies, ligustrazine has shown great potential in the treatment of neuroinflammation-related disorders, and it can significantly relieve some types of neuropathic pain [56]. In this paper, we confirmed that ligustrazine effectively relieved migraine-like pain behaviors. Most importantly, ligustrazine also inhibited ROS overproduction and alleviated decreased mitochondrial potential, emphasizing the important role of TCM antioxidative therapies in the management of migraine headaches. Although ligustrazine has shown great potential in relieving migraine and neuroinflammation, the clinical treatment of migraine is mostly based on the use of mixed formulations of several Chinese herbs, mainly *Chuanxiong*, *Astragalus*, *Tianma*, *etc*., and there have been reports suggesting synergistic or facilitating effects between ligustrazine and other Chinese herbal ingredients [57,58]. Therefore, in this study, we focused only on ligustrazine itself and ignored other herbal components, which is another limitation of this work. Moreover, our further results proved that D2R is a candidate pharmacological target for ligustrazine using molecular docking. The energy values calculated by molecular docking are different for each docking time, with smaller values being considered more significant. Our results showed that the best value was −4.24 kcal/mol, which confirmed the effective but not powerful interaction between D2R and ligustrazine. Therefore, further evaluations of combinations of multiple herbal ingredients and the role played by D2R remain essential.

## 5. Conclusions

In conclusion, the GluA2 distribution at the synapses was disrupted, and GluA2 endocytosis increased the intracellular calcium and mitochondrial calcium overload and ROS overproduction, which, in turn, enhanced GluA2 endocytosis. D2R activation blocked GluA2 internalization, enabling GluA2 to be recirculated on the synaptic surface and effectively alleviating pain hypersensitivity in CM rats. Ligustrazine and drugs targeting D2R could be effective options for treating CM.

## Figures and Tables

**Table 1:** IS information.

Component	Manufacturer	Concentration
Bradykinin	Sigma-Aldrich, St. Louis, MO, USA	1 mM
Histamine	Sigma-Aldrich, St. Louis, MO, USA	1 mM
Serotonin	Sigma-Aldrich, St. Louis, MO, USA	1 mM
Prostaglandin E2	Sigma-Aldrich, St. Louis, MO, USA	0.1 mM

**Table 2:** Antibody information.

Antibody	Manufacturer	Dilution
Anti-GluA2	Abcam, Cambridge, UK	1:1000
	Proteintech, Wuhan, China	1:1500
Anti-N-cadherin	PTM Biolabs, Hangzhou, China	1:1000
GAPDH	ZEN-BIOSCIENCE, Chengdu, China	1:8000
Anti-D2R	Proteintech, Rosemont, IL, USA	1:1000
Anti-CGRP (For IF)	Santa Cruz, Santa Cruz, CA, USA	1:400
Goat anti-rabbit IgG	ZEN-BIOSCIENCE, Chengdu, China	1:5000
Goat anti-mouse IgG	ZEN-BIOSCIENCE, Chengdu, China	1:5000
Alexa Fluor 488 anti-mouse IgG	Beyotime, Haimen, China	1:400
Cy3-labeled goat anti-mouse IgG	Beyotime, Haimen, China	1:400

**Table 3:** The best binding energy values (top 5 of 50 runs).

Binding Energy (kcal/mol)
−4.24
−4.23
−4.08
−3.96
−3.91

## Data Availability

The datasets used and analyzed during the current study are available from the corresponding author on reasonable request.

## Supplementary Materials

The following supporting information can be downloaded at: https://www.mdpi.com/article/10.3390/antiox13060725/s1, Figure S1: The effect of quinpirole and ligustrazine on ROS level under normal conditions. (**A**), quinpirole and ligustrazine did not affect normal ROS production in neurons. Scale Bar = 50 μm. Data are mean ± SEM (*n* = 3/group). ns, not significant. (**B**), both quinpirole and ligustrazine did not affect normal mitochondrial membrane potential in neurons. Scale Bar = 20 μm. Data are mean ± SEM (*n* = 3/group). ns, not significant. (**C**), quinpirole and ligustrazine have similar roles in mitigating L-AMPA-induced ROS generation. Scale Bar = 50 μm. Data are mean ± SEM (*n* = 3/group). *** *p* < 0.001 vs. the DMSO group; ### *p* < 0.001 vs. the L-AMPA group; ns, not significant.

## Abbreviations

AMPARs	α-Amino-3-hydroxy-5-methyl-4-isoxazole propionic acid receptors
CM	Chronic migraine
CNS	Central Nervous System
D2R	Dopamine D2 receptor
D3R	Dopamine D3 receptor
IS	Inflammatory soup
LCH	Ligusticum chuanxiong hort
PFA	Paraformaldehyde
ROS	Reactive oxygen species
TNC	Trigeminal nucleus caudalis
