# Supplementary figures and images for: Dopamine D2 Receptor Activation Blocks GluA2/ROS Positive Feedback Loop to Alienate Chronic-Migraine-Associated Pain Sensitization
Source: Antioxidants (Basel). 2024 Jun 14;13(6):725. doi: 10.3390/antiox13060725 (PMC11201052; doi:10.3390/antiox13060725)

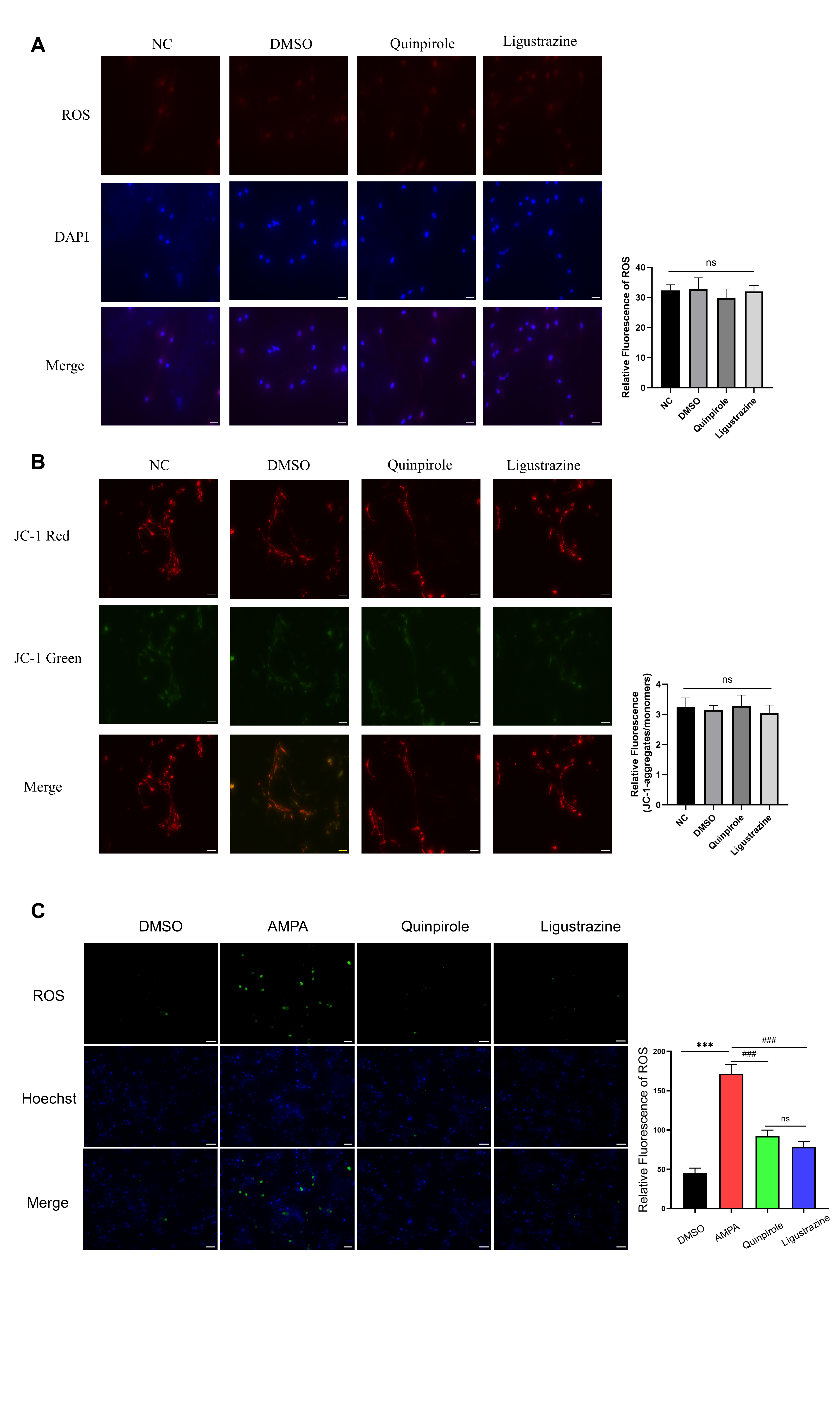

Supplement: Supplementary file 1 [file antioxidants-13-00725-s001.zip › antioxidants-2991374-supplementary.tif]
